# Pathogenicity and Comparative Genomics Analysis: *Pasteurella multocida* Serogroups A and F Isolated from Domestic Rabbits in China

**DOI:** 10.3390/vetsci13101058

**Published:** 2026-10-08

**Authors:** Qiaoya Zhao, Cunxia Liu, Xiuli Ma, Feng Hu, Junfeng Lv, Tong Zhu, Yajian Gao, Changhao Liu, Bing Huang, Yifei Jiang

**Affiliations:** 1 Poultry Institute, Shandong Academy of Agricultural Sciences, Jinan 250100, China; zqya1990@163.com (Q.Z.);; 2 Shandong Provincial Key Laboratory of Livestock and Poultry Breeding, Jinan 250100, China; 3 College of Veterinary Medicine, Inner Mongolia Agricultural University, Hohhot 010011, China; 4 Qilu Animal Health Products Co., Ltd., Jinan 250100, China

**Keywords:** *Pasteurella multocida*, whole-genome sequencing, pathogenicity, serogroup A, serogroup F

## Abstract

*Pasteurella multocida* is a zoonotic pathogen that causes severe respiratory infections in domestic rabbits. Serogroup F strains have been increasingly reported but remain poorly characterized. Two strains, PS-JQ08 (serogroup A) and PS-JQ09 (serogroup F), were isolated from deceased rabbits in China. Pathogenicity was assessed in mouse and rabbit models, and whole-genome sequencing was performed for comparative genomic analysis. Both strains were highly pathogenic but induced distinct pathological profiles: PS-JQ08 caused hepatocellular vacuolar degeneration, whereas PS-JQ09 induced hemorrhage and edema. Genomic analysis revealed that both strains harbored core virulence genes involved in adhesion, iron acquisition, and antiphagocytosis; however, the serogroup F strain harbored unique iron transport genes and arsenic resistance systems. Phylogenetic analysis revealed that PS-JQ08 clustered with avian serogroup A strains, whereas PS-JQ09 clustered with porcine- and rabbit-derived serogroup F strains. These findings provide clues for exploring the genetic mechanism underlying pathogenic differentiation of *Pasteurella multocida* between serogroups A and F, and provide valuable genomic information to support vaccine development and disease control in rabbits.

## Simple Summary

*Pasteurella multocida* is a major pathogen responsible for respiratory infections in domestic rabbits. Serogroup F strains have been reported in recent years but remain poorly characterized. In this study, two highly virulent strains belonging to serogroups A and F, were isolated from a diseased rabbit farm. Animal infection experiments and whole-genome sequencing were performed to compare their pathogenicity and genomic characteristics. Both strains caused mortality in experimentally infected animals but produced distinct patterns of tissue damage, The serogroup F strain also harbored unique genes associated with iron uptake and immune evasion. These findings provide fundamental data for the diagnosis, treatment, and vaccine development against rabbit pasteurellosis, and are of great significance to the sustainable development of the rabbit industry.

## 1. Introduction

*Pasteurella multocida* (*P. multocida*) is a Gram-negative, facultatively anaerobic zoonotic pathogen that infects a wide range of domestic animals, wildlife, and humans, causing diseases such as hemorrhagic septicemia, fowl cholera, atrophic rhinitis in swine, and pneumonia in cattle and sheep [1,2,3,4]. Based on capsular antigen differences, *P. multocida* is classified into five capsular serogroups (A, B, D, E, and F), which are primarily associated with respiratory tract infections [5,6]. Rabbits are highly susceptible to *P. multocida*, which can causes rhinitis, pneumonia, otitis media, and septicemia, often resulting in high mortality and substantial economic losses to the rabbit industry [7,8,9]. Although serogroup A strains are currently predominant among rabbits in China, reports of serogroup F isolates have increased in recent years, highlighting their growing epidemiological significance [4,9,10]. However, whole-genome data on rabbit-derived *P. multocida*, particularly serogroup F strains, remain scarce compared with those available for isolates from swine, poultry, and cattle [11,12]. Consequently, the pathogenic mechanisms, antimicrobial resistance profiles, and genetic evolution of these strains remain poorly understood. Whole-genome sequencing has been widely applied to molecular, epidemiological, and mechanistic studies of *P. multocida* [13]. For example, a comparative genomic analysis of bovine-derived serogroup B strains from Pakistan revealed the frequent absence of the protective antigens OmpH and PlpE [14]. Similarly, the first genome sequence of serogroup A strain SHZ01, isolated from hybrid sheep in China, revealed 80 type III secretion system effector proteins and 54 antimicrobial resistance genes [15]. These findings demonstrate substantial variation in genomic architecture, virulence-associated gene profiles, and resistance spectra among strains from different hosts and serogroups.

Nevertheless, most previous comparative genomic studies focused on strains from bovine, ovine, avian or porcine hosts, and few studies have jointly compared pathogenic phenotypes and genomic features between matched serogroup A and F isolates from rabbits. In this study, two *P. multocida* strains were isolated from the lungs of naturally deceased rabbits at a large-scale rabbit farm in Shandong Province, China, and were identified as capsular serogroups A and F. Using pathogenicity assays in mice and rabbits, whole-genome sequencing, and comparative genomic analysies, we systematically characterized the genomic features, virulence-associated gene genealogies of the two strains were systematically elucidated. Their pathogenicity was further evaluated in vivo, revealing distinct disease phenotypes: the serogroup A strain induced pneumonia, whereas the serogroup F strain caused hemorrhagic manifestations. These findings provide a basis for the subsequent development of vaccines and diagnostic reagents, as well as antimicrobial resistance surveillance of rabbit-derived *P. multocida*.

## 2. Materials and Methods

### 2.1. Ethical Statement

The animal experimental protocol used in this study was approved by the Ethics Committee for Animals of the Shandong Academy of Agricultural Sciences, China. All procedures were conducted in accordance with the Guidelines for the Management and Use of Laboratory Animals.

### 2.2. Sample Source and Bacterial Isolation

Pathological samples were collected from naturally deceased rabbits at a large-scale commercial rabbit farm in Shandong Province, China. The rabbits had not received antibiotic treatment before sampling. Clinical signs observed before death included reduced appetite, nasal discharge, and dyspnea. Lung tissues with obvious lesions were aseptically collected and immediately processed for bacterial isolation.

The cut surface of each lung tissue sample was streaked onto Luria–Bertani (LB) agar plates supplemented with 5% newborn bovine serum using an inoculation loop. The plates were incubated at 37 °C for 16–24 h. Single colonies were selected and sub-cultured three consecutive times. Colonies from the third passage were selected for large-scale culture and subsequent experiments. Purified isolates were subjected to Gram staining and microscopic examination. Genomic DNA was extracted using a Bacterial Genomic DNA Extraction Kit (Solarbio, Beijing, China, Catalog No. D1600) and stored at −20 °C until further analysis.

### 2.3. Species and Serogroup Identification

Species-specific identification of *P. multocida* was performed by amplifying the kmt1 using the primers KMT1SP6/KMT1T7 primers, as described by Townsend et al. [16]. Capsular serotyping was performed using a multiplex PCR assay targeting the following gene fragments: hyaD-hyaC (serogroup A), bcbD (serogroup B), dcbF (serogroup D), ecbJ (serogroup E), and fcbD (serogroup F). LPS genotyping was performed using the LPS multiplex polymerase chain reaction (PCR) typing method established by Harper et al. [17].

### 2.4. Animal Pathogenicity Assays

To comprehensively evaluate the pathogenicity of the two rabbit-derived *P. multocida* strains, mouse and rabbit infection models were established to assess acute systemic and respiratory infection, respectively.

#### 2.4.1. Mouse Pathogenicity Assay

Fifteen 6-to 8-week-old SPF Kunming mice were randomly assigned to three groups (n = 5 per group): PS-JQ08 challenge, PS-JQ09 challenge, and negative control groups. Overnight-activated cultures of each bacterial strain were centrifuged, resuspended, and their concentration was adjusted to 1 × 10^9^ colony-forming units/mL (CFU)/mL. Each mouse was intraperitoneally injected with 0.2 mL of the bacterial suspension (2 × 10^8^ CFU), whereas mice in the control group received an equal volume of sterile phosphate-buffered saline (PBS). The mice were continuously monitored for 72 h post-challenge. Mental status, feeding and drinking behavior, and time of death were recorded. Mice that died during the observation period were immediately necropsied and gross lesions in the lungs, liver, and spleen were examined. Surviving mice were euthanized at 72 h. Lung and liver tissues were collected, fixed in 4% paraformaldehyde, embedded in paraffin, sectioned, and stained with hematoxylin and eosin (H&E) for histopathological examination under a light microscope.

#### 2.4.2. Rabbit Pathogenicity Assay

Fifteen 8-to 10-week-old SPF New Zealand White rabbits were randomly assigned to three groups (n = 5 per group): PS-JQ08, PS-JQ09, and negative control. Bacterial suspensions were prepared as described above and adjusted to a final concentration of 5 × 10^9^ CFU/mL. For intranasal inoculation 1 mL of the bacterial suspension (5 × 10^9^ CFU) was instilled into the nostrils of each rabbit. Rabbits in the control group received an equal volume of sterile PBS. The rabbits were monitored for 14 days post-challenge, and respiratory rate, general condition, feed intake, and mortality were recorded. Rabbits that became moribund or died were immediately necropsied, and lesions in the trachea, lungs, heart, liver, spleen, and kidneys were examined. The surviving rabbits were euthanized on day 14. Lung, liver, spleen, and kidney tissues were collected for histopathological examination (hematoxylin and eosin staining) and bacterial isolation.

### 2.5. Genome Sequencing and Analysis

High-quality genomic DNA was extracted from both bacterial strains using the NucleoBond^®^ HMW DNA kit (MN NucleoBond, Düren, Germany, Cat. No. 740160.20). DNA concentration was quantified with a Qubit 4.0 fluorometer (Thermo Fisher Scientific, Waltham, MA, USA, Cat. No. Q33226), while DNA purity was assessed using a NanoDrop SMA4000 spectrophotometer (Thermo Fisher Scientific, Waltham, MA, USA). DNA integrity was verified by 0.75% agarose gel electrophoresis. The extracted genomic DNA was divided into two aliquots. One aliquot was randomly fragmented to construct an Illumina library with an insert size of 300 bp, which was subjected to paired-end 150 bp sequencing on the Illumina NovaSeq 6000 platform. The other aliquot was processed directly through end repair, 3′ adenylation, and ligation of sequencing adapters together with motor proteins. The ligation products were purified using Agencourt AMPure XP Beads (Beckman Coulter, Brea, CA, USA, Cat. No. A63881). Fragments longer than 1 kb were subsequently enriched for single-molecule nanopore DNA sequencing using a MinION Flow Cell (ONT, Oxford, UK, R9.4.1).

Raw nanopore reads were filtered and assembled with Canu using default parameters. The resulting genomic sequences were polished with NextPolish (v1.4.1) and Pilon (v1.18). Gene prediction and functional annotation were performed using Prokka (v1.10). Predicted protein sequences were searched against the NCBI nr database. Functional classification of protein-coding genes was implemented based on the Cluster of Orthologous Groups of proteins and the Kyoto Encyclopedia of Genes and Genomes databases.

Potential virulence-associated genes were predicted using BLAST+ (version 2.16.0) BLASTp against the core dataset of the Virulence Factor Database (VFDB), and antibiotic resistance genes (ARGs) were screened using the Comprehensive Antibiotic Resistance Database (CARD) with the thresholds of E-value ≤ 1 × 10^−5^, identity ≥ 40%, and coverage ≥ 40%. Additionally, comparative analysis of the capsule locus and the LPS outer core biosynthesis region were performed.

## 3. Results

### 3.1. Bacterial Isolation and Identification

**Figure 1:**
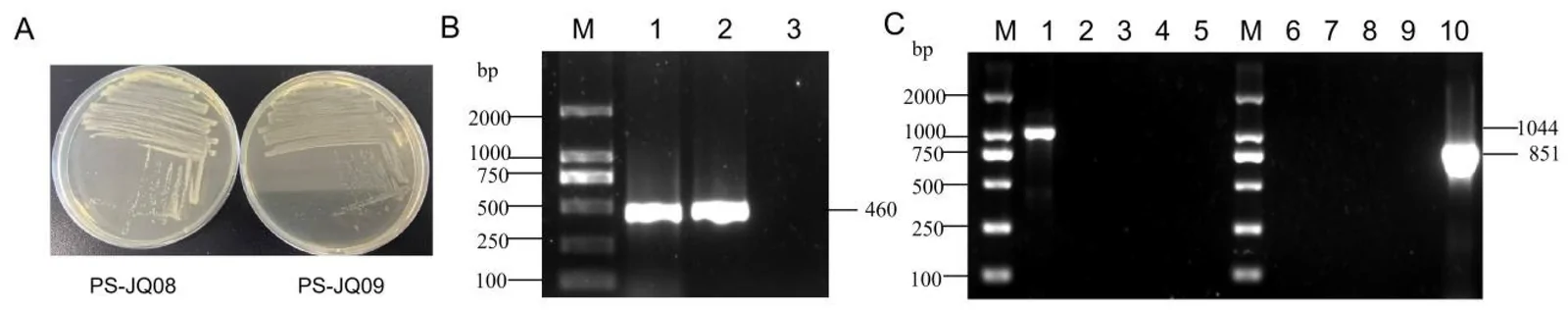
Identification of *Pasteurella multocida* isolates PS-JQ08 and PS-JQ09. (**A**) Colony morphology on serum-supplemented agar. (**B**) Amplification products of the kmt1 gene using Pasteurella species-specific primers. (**C**) Amplification products using *P. multocida* serogroup-specific primers.

After incubation at 37 °C for 24 h on LB agar supplemented with 5% serum, two morphologically distinct colony types of grayish-white, translucent, round, and smooth colonies were isolated from the lung samples of deceased rabbits (Figure 1A). Both colony types were grayish-white, translucent, round, and smooth. Colonies of strain PS-JQ08 were approximately 1.5–2.0 mm in diameter with smooth margins, whereas those of strain PS-JQ09 were approximately 1.0–1.2 mm in diameter and slightly mucoid. Gram staining revealed that both strains were Gram-negative short rods, occurring singly or in pairs.

Species-specific PCR results indicated that both strains amplified an approximately 460 bp fragment of the kmt1 gene in both strains, confirming their identification as *P. multocida* (Figure 1B). Capsular serotyping by multiplex PCR showed that PS-JQ08 amplified only the 1044 bp hyaD-hyaC fragment, identifying it as serogroup A, whereasPS-JQ09 amplified only an 851 bp fcbD fragment, identifying it as serogroup F (Figure 1C). The two isolates were identified as LPS genotypes L6 and L3, respectively.

### 3.2. Animal Pathogenicity

#### 3.2.1. Pathogenicity in Mice

To evaluate the pathogenicity of the two Pasteurella isolates, mice were intraperitoneally inoculated infected with 2 × 10^8^ CFU. All mice in the negative control group remained asymptomatic and showed no clinical signs throughout the observation period. In contrast, mice in both infection groups developed lethargy and depression, within 6 h post-inoculation. Mortality subsequently occurred, with the majority of the infected mice succumbing within 12 h post-inoculation. All mice in the PS-JQ08 group died within 72 h (Mortality and sampling information of mice are provided in the Supporting Information: Table S1).

**Figure 2:**
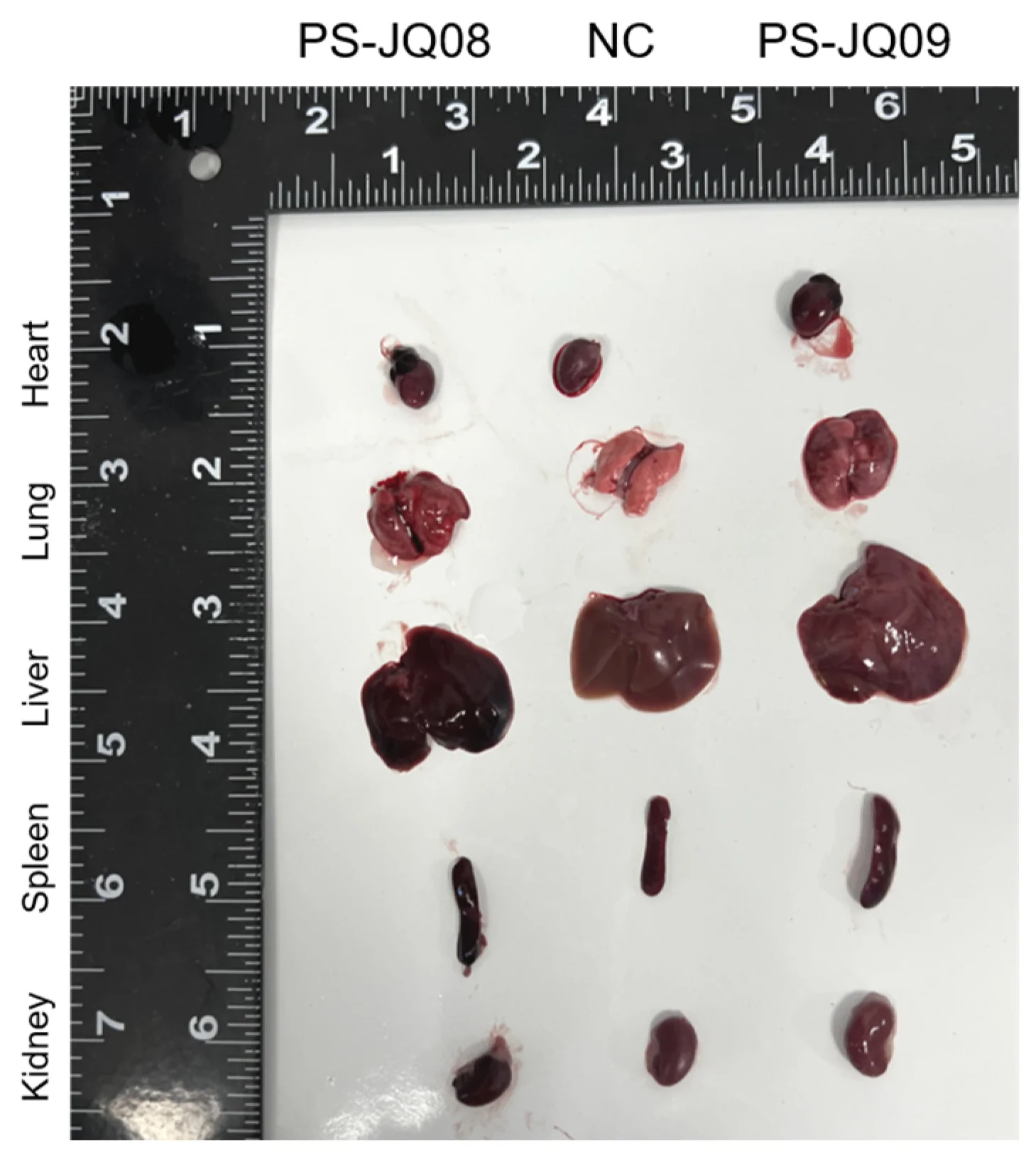
Gross pathological changes in the mice. Left: PS-JQ08-infected mouse; middle: healthy control mouse; right: PS-JQ09-infected mouse.

Necropsy results showed that no obvious pathological changes were observed in various organs of the control mice. In contrast, mice from the infected groups exhibited severe hemorrhages in the lungs and liver as well as splenomegaly (Figure 2). No significant changes were observed in the ovaries, testes, or other organs. Control mice showed no clinical symptoms or mortality.

**Figure 3:**
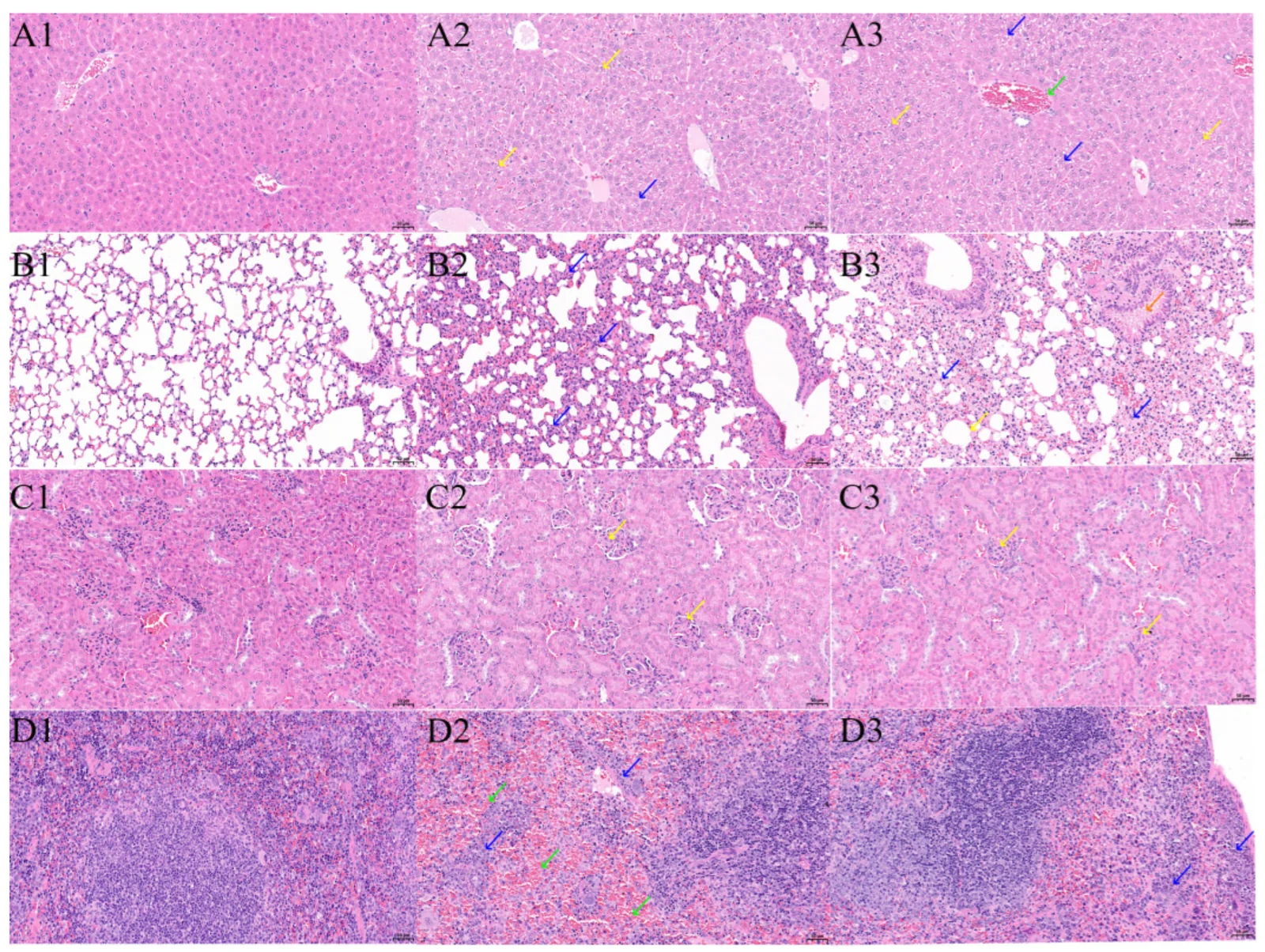
Histopathological observation of multiple organs (H&E staining). (**A**) Liver: (**A1**) Control. (**A2**) PS-JQ08: Numerous hepatocytes with vacuolar degeneration (yellow arrows) and a few with hydropic degeneration (blue arrows). (**A3**) PS-JQ09: Vacuolar (yellow arrows) and more extensive hydropic degeneration (blue arrows), with mild vascular congestion (green arrows). (**B**) Lung: (**B1**) Control. (**B2**) PS-JQ08: Extensive granulocytic infiltration in the alveolar walls (blue arrows), with mild alveolar wall thickening and septal widening. (**B3**) PS-JQ09: Bronchiolar eosinophilic material (orange arrows), focal hemorrhage, scattered granulocytes in alveolar walls (blue arrows), moderate septal thickening, and compensatory emphysema in a few alveoli (yellow arrows). (**C**) Kidney: (**C1**) Control. (**C2**) PS-JQ08 and (**C3**) PS-JQ09: Both strains caused mild glomerular mesangial hyperplasia (yellow arrows) in a few glomeruli. (**D**) Spleen: (**D1**) Control. (**D2**) PS-JQ08: Numerous extramedullary hematopoietic cells around the trabeculae (blue arrows) and sinusoidal congestion (green arrows). (**D3**) PS-JQ09: Aggregates of extramedullary hematopoietic cells around the capsule and trabeculae (blue arrows). Scale bars: 50 μm.

Histopathological examination confirmed the presence of *P. multocida*-associated lesions. No pathological changes were detected in the negative control groups (Figure 3(A1,B1,C1,D1)). In the PS-JQ08 group, lesions were predominantly observed in the lungs and were characterized by thickening of the interlobular septa, marked inflammatory cell infiltration, and serous and fibrinous exudates within the alveolar spaces (Figure 3(B2)). Mild lesions were observed in the kidneys and spleen (Figure 3(C2)), whereas the hepatocytes primarily exhibited vacuolar degeneration (Figure 3(A2)). In the PS-JQ09 group, the most severe lesions were observed in the liver and were characterized by hepatocellular edema, cellular swelling, pale and loosely arranged, and hepatic sinusoidal congestion (Figure 3(A3)). In the lungs, interlobular septal thickening and marked inflammatory cell infiltration were accompanied by eosinophilic material within the bronchioles and alveolar hemorrhage (Figure 3(B3)). In both groups, numerous extramedullary hematopoietic cells were aggregated around the splenic trabeculae (Figure 3(D2,D3)).

#### 3.2.2. Pathogenicity in Rabbits

Within 12 h post-challenge, all rabbits in the challenge groups developed hyperthermia (≥40.5 °C), which persisted for 72 h. Body temperatures in the negative control group remained below 39.5 °C throughout the observation period. Clinical signs included depression, anorexia, tachypnea (60–80 breaths/min), and serous or purulent nasal discharge. Some rabbits developed open-mouth breathing and mouth and nose cyanosis (Mortality and sampling information of rabbit are provided in the Supporting Information: Table S2).

**Figure 4:**
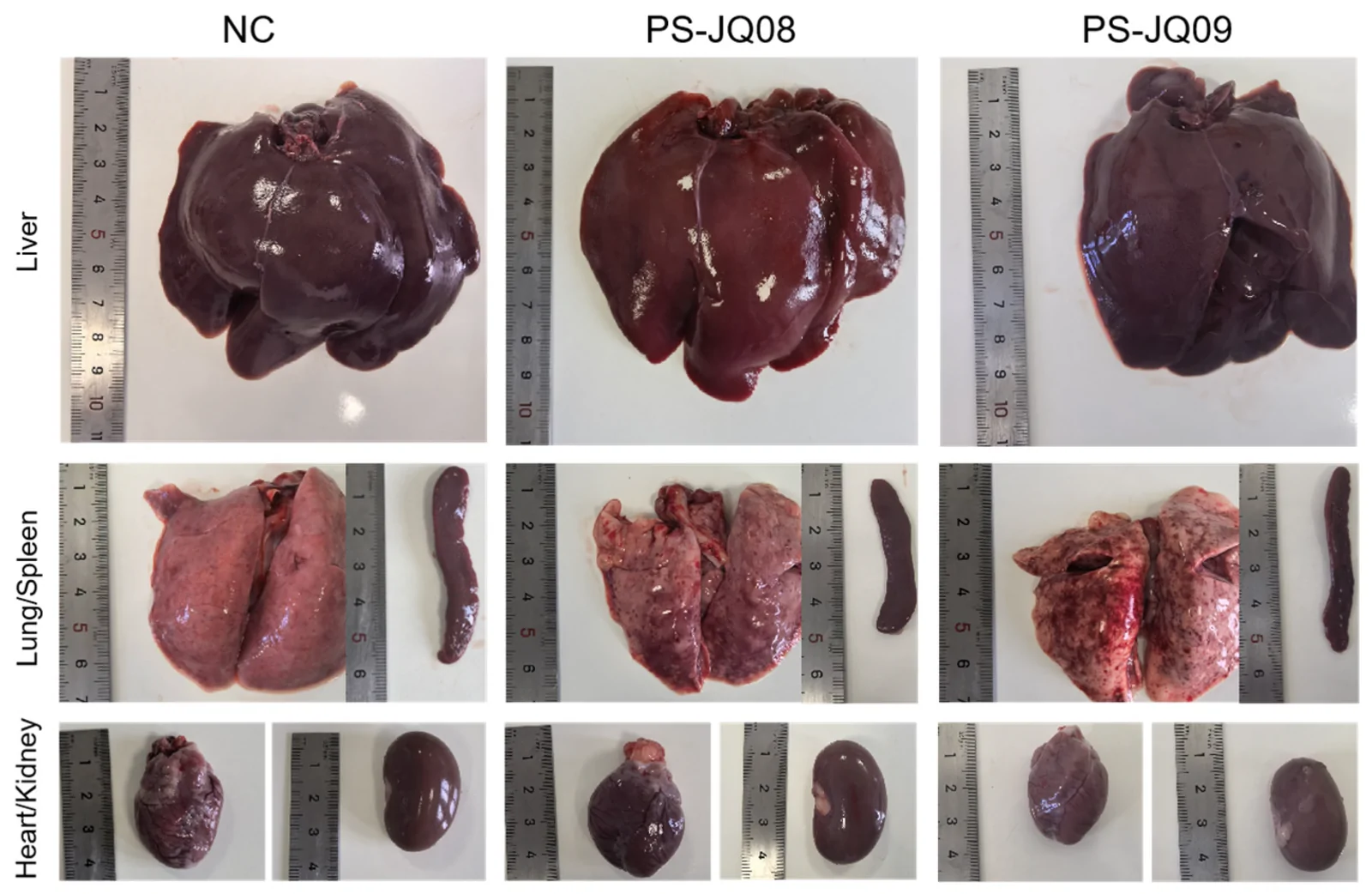
Gross pathological lesions in rabbits infected with *Pasteurella multocida* strains PS-JQ08 and PS-JQ09. Sampling was collected from major organs (heart, liver, lungs, spleen, and kidneys).

Necropsy revealed systemic lesions in all challenged rabbits, consistent with the characteristics of pasteurellosis (Figure 4). The lungs were pale and shrunken and exhibited grayish-white nodules, severe congestion, and diffuse consolidation. Adhesions between the lung lobes and thoracic wall were observed. Pericardial effusion and petechial hemorrhages were present on the epicardium. The liver was mildly enlarged and congested, while the spleen was mildly enlarged, displaying a purplish-black color and a brittle texture. In the control group, the lungs were pink and elastic, and all organs exhibited normal histological morphology. The inoculated strains were successfully re-isolated from the liver and blood of all rabbits in the challenge groups that died or reached a moribund state. PCR confirmed that the re-isolated strains were identical to the corresponding challenge strains. All organ samples from the control group tested negative.

**Figure 5:**
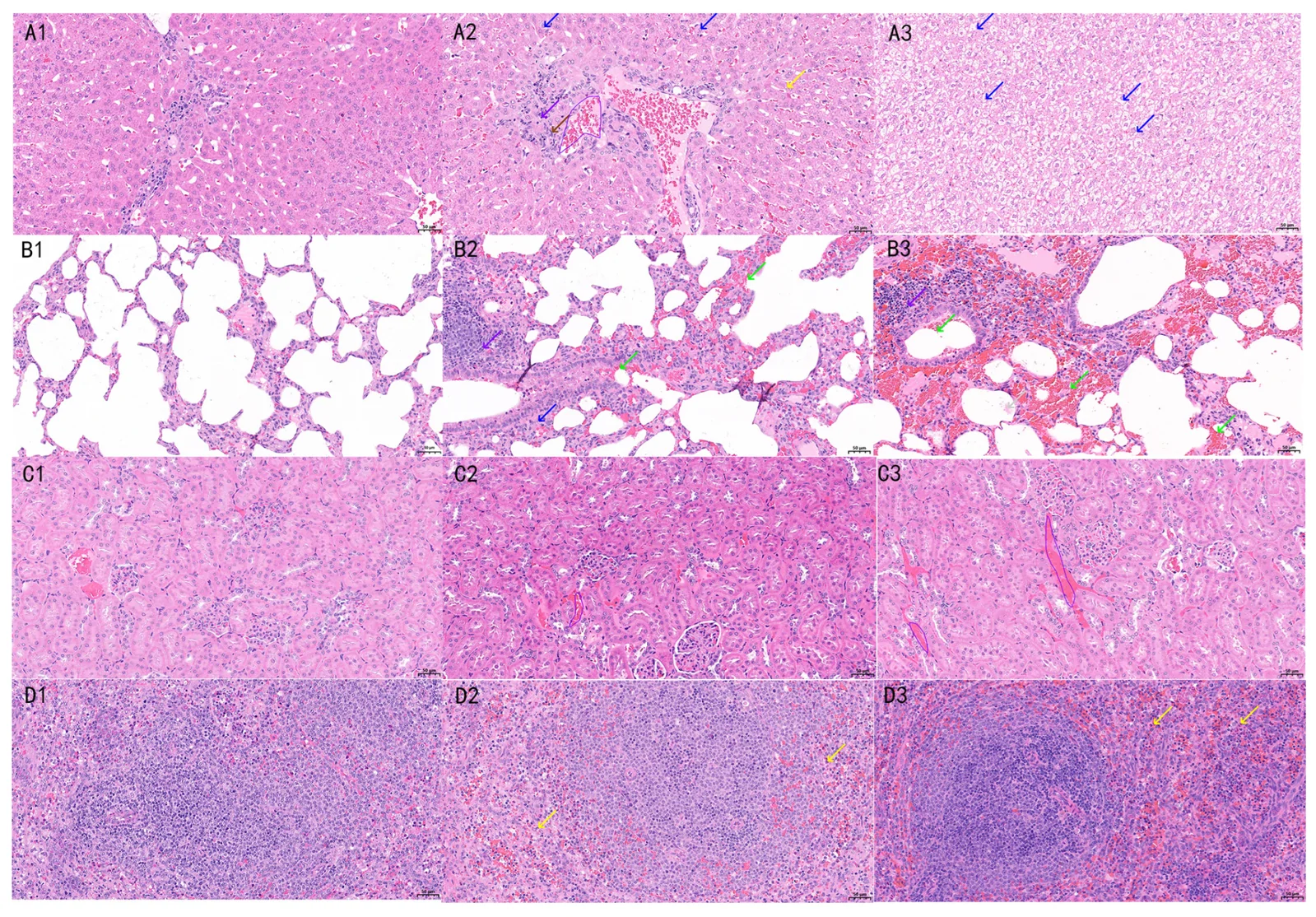
Histopathological findings in rabbits infected with *Pasteurella multocida* strains PS-JQ08 and PS-JQ09. Samples were collected from major organs (liver, lung, spleen, and kidney) at 7 days post-challenge or when humane endpoint criteria were met. (**A**) Liver: (**A1**) Control. (**A2**) PS-JQ08: Notable hepatocyte vacuolar degeneration with small, round cytoplasmic vacuoles (yellow arrows), occasional hepatocyte edema (blue arrows), and mild hepatic sinusoidal congestion (purple polygons), slight proliferation of connective tissue around the portal area (brown arrows), with occasional lymphocyte infiltration (purple arrows). (**A3**) PS-JQ09: notable hepatocyte edema (blue arrows). (**B**) Lung: (**B1**) Control. (**B2**) PS-JQ08: Granulocytic infiltration of the alveolar walls (blue arrows), mild thickening of the alveolar walls over a relatively large area, widening of the alveolar septa, and focal hemorrhage (green arrows); focal or aggregated lymphocyte infiltration around a few blood vessels and bronchioles (purple arrows). (**B3**) PS-JQ09: Small amounts of eosinophilic material in the alveoli (gray arrows), extensive bronchiolar and alveolar hemorrhage (green arrows), and focal mild thickening of the alveolar walls with widening of the alveolar septa, occasional focal lymphocyte infiltration around blood vessels and bronchioles (purple arrows). (**C**) Kidney: (**C1**) Control. (**C2**) PS-JQ08: Mild vascular congestion (purple polygons). (**C3**) PS-JQ09: Notable vascular congestion (purple polygons) and a mild increase in glomerular matrix. (**D**) Spleen: (**D1**) Control. (**D2**) PS-JQ08: Sinusoidal congestion in the red pulp (yellow arrows) and scattered granulocytic infiltration. (**D3**) PS-JQ09: Widespread aggregation of extramedullary hematopoietic cells (yellow arrows) and scattered granulocytic infiltration. Scale bars: 50 μm.

Histopathological examination further revealed hemorrhage and congestion of varying degrees in both the PS-JQ08 and PS-JQ09 groups (Figure 5). Diffuse fibrinous suppurative pneumonia was observed in the lungs, characterized by extensive infiltration of neutrophils and macrophages within the alveolar spaces and fibrinous exudation. The alveolar septa were significantly widened. Necrosis and sloughing of the bronchiolar epithelium were also observed, accompanied by inflammatory cells and exudates within the bronchiolar lumen. Hepatic lesions were consistent with those observed in the mouse model. The PS-JQ08 group primarily exhibited vacuolar degeneration, whereas the PS-JQ09 group exhibited hepatocellular edema. Foci of coagulative necrosis were observed in some areas, accompanied by sinusoidal dilation and congestion. In the spleen, white pulp atrophy with indistinct architecture, red pulp congestion, and macrophage hyperplasia were observed.

### 3.3. Basic Genomic Characteristics

**Figure 6:**
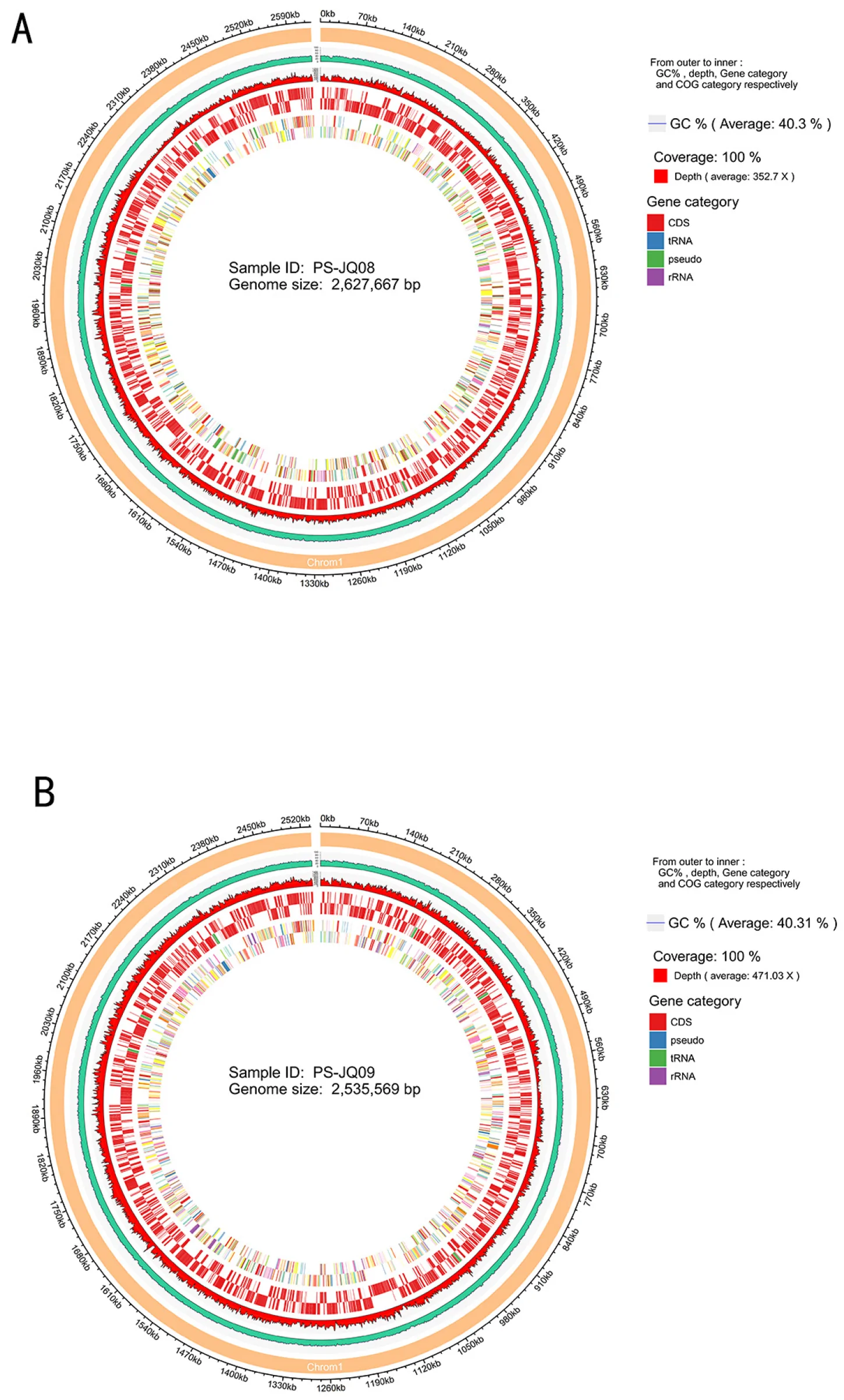
Genomic Circos diagrams of *Pasteurella multocida* strains PS-JQ08 (**A**) and PS-JQ09 (**B**). From outer to inner, the circular diagrams display GC content, sequencing depth, genetic elements, and COG functional classification.

The read length distribution of sequencing reads for the two strains is illustrated in Supporting Information: Figure S1, Sequencing and assembly statistics for the two strains are presented in Supporting Information: Tables S3 and S4. For PS-JQ08, genome sequencing generated 9,111,574 reads with a total read length of 1,355,789,277 bp, providing an approximately 352.7-fold coverage of the entire genome. Similarly, for PS-JQ09, 11,021,292 reads were generated with a total read length of 1,644,260,629 bp, achieving high coverage of approximately 471.03-fold. For PS-JQ08 and PS-JQ09, the assembled genome sizes were 2,627,667 bp and 2,535,569 bp, and their GC contents were 42.36% and 42.24%, respectively. Genome annotation predicted 2615 genes in PS-JQ08 and 2509 genes in PS-JQ09. These predicted genes were further characterized, revealing 2492 protein-coding sequences (CDS) for PS-JQ08 and 2383 for PS-JQ09. Circular genome maps for PS-JQ08 and PS-JQ09 were generated using Circos software, and the single-ring genomic maps of the two *P. multocida* strains are shown in Figure 6. Overall, only minor differences were observed between PS-JQ08 and PS-JQ09 in terms of genome size, predicted gene number, and G + C content.

### 3.4. Genomic Mobile Elements

Eight genomic islands (GIs) were predicted for each strain; however, their gene compositions differed substantially. The GIs shared by both strains contained genes encoding ssb (single-stranded DNA-binding protein), rrf (ribosome recycling factor), mukB (chromosome segregation protein), and phage-related proteins (gpJ and gpU). GIs unique to PS-JQ08 harbored genes involved in amino acid and lipid metabolism, such as phosphatidylglycerophosphate synthase (pgsA) and aspartate kinase (lysC). In contrast, GIs unique to PS-JQ09 contained genes associated with heavy-metal resistance, including arsB and arsC, which are components of arsenic-resistance systems (Supporting Information: Figure S2).

In PS-JQ08, the genes identified within the GIs were primarily associated with phage structure (gpJ + gpU) and membrane/cell wall biosynthesis (pgsA and gmhB). These genomic features may contribute to strain-specific differences in membrane structure or LPS antigenicity.

In PS-JQ09, the GIs exhibited more diverse functional profiles. In addition to phage-associated elements, some GIs contained genes involved in arsenic resistance and DNA repair or regulation. These features may contribute to the strain’s ability to survive under environmental stress conditions, including exposure to heavy metals and oxidative stress.

Both strains contained gmhB, a gene involved in LPS biosynthesis, suggesting a potentially shared capacity for modifying LPS-associated surface properties. PS-JQ08 harbored seven intact prophage regions ranging from 8.1 to 125.7 kb, whereas PS-JQ09 harbored six intact prophage regions, ranging from 16.2 to 116.1 kb (Table 1). Some prophage regions contained genes associated with metabolic adaptation and virulence.

### 3.5. Genome Annotation

**Figure 7:**
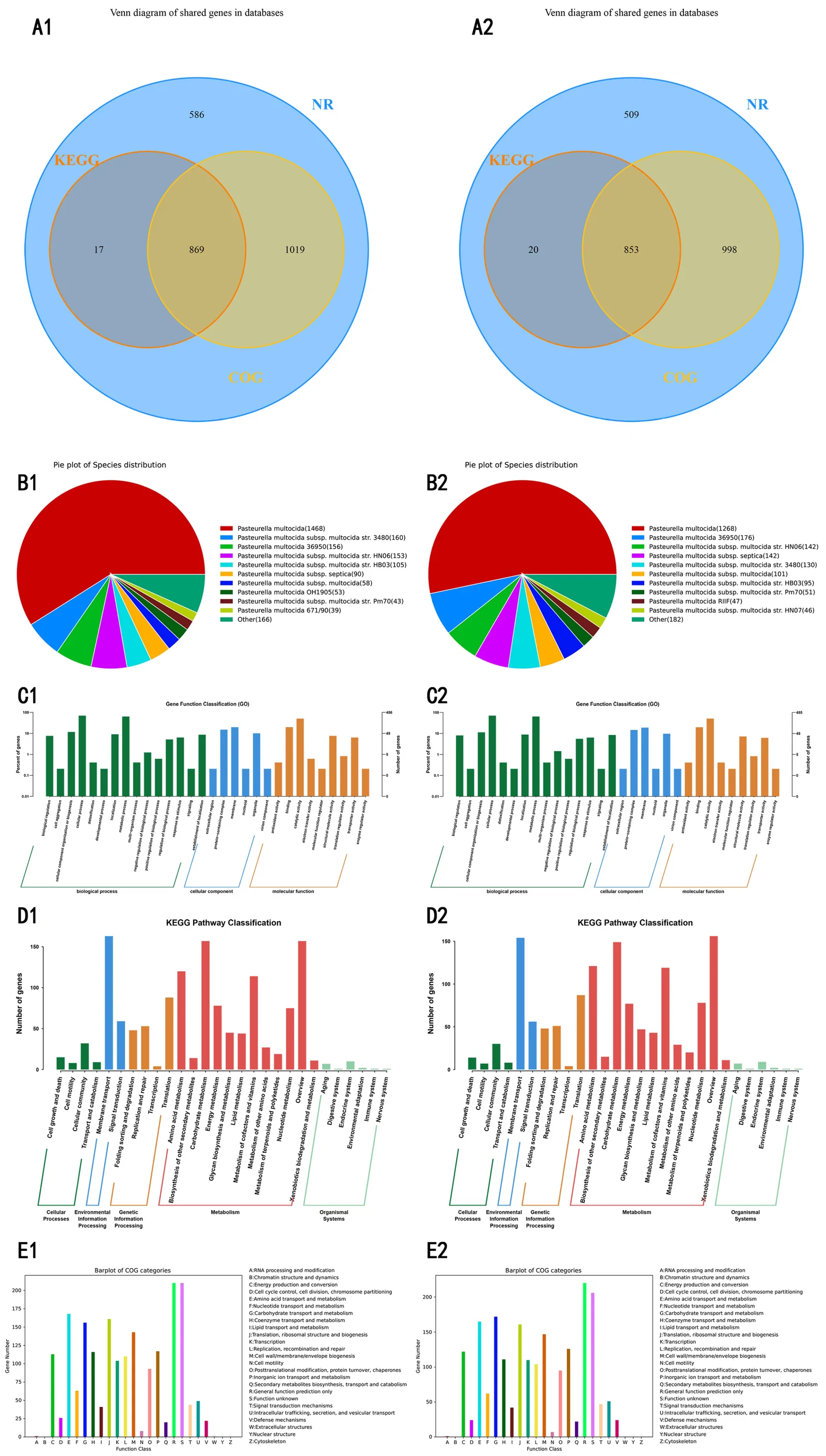
Functional annotation of *Pasteurella multocida* strains PS-JQ08 and PS-JQ09. (**A**) Venn diagram of genes annotated in the databases. (**B**) Pie chart of homologous species distribution: each sector in the chart represents a species, and a larger sector area indicates a greater number of sequences aligned to that species. (**C**) GO annotation distribution bar chart: the horizontal axis represents the secondary GO classifications, and the vertical axis represents the number of genes within each classification and their percentage of the total annotated genes. Different colors represent different orthologs. (**D**) KEGG Pathway classification bar chart: the horizontal axis represents the names of the metabolic pathways involved, and the vertical axis represents the number of genes annotated to each pathway. (**E**) COG classification bar chart: the horizontal axis, with each color representing a functional category of COG, and the vertical axis representing the number of genes annotated to each category. (**1**), strain PS-JQ08; (**2**), strain PS-JQ09.

The genomes of PS-JQ08 and PS-JQ09 contained 2492 and 2383 coding sequences (CDSs), respectively, of which 99.96% (PS-JQ08) and 99.87% (PS-JQ09) showed homology in the NR database. Annotation rates for COG, KEGG, GO, and CAZy were 75.76% vs. 77.68%, 35.55% vs. 36.63%, 19.50% vs. 20.35%, and 1.24% vs. 1.22%, respectively. In the virulence and resistance databases, annotations were found for PHI (22.51% vs. 22.95%), VFDB (5.90% vs. 6.00%), and CARD (2.37% vs. 2.48%). A total of 869 and 853 core genes were shared among the NR, KEGG, and COG annotations of the two strains (Figure 7(A1,A2)). Comparative genomic analysis of the NR database revealed the highest homology with *P. multocida* 3480 (160 genes, serogroup A, porcine origin) for PS-JQ08, and with *P. multocida* 36950 (176 genes, bovine HS origin) for PS-JQ09 (Figure 7(B1,B2)).

Gene Ontology (GO) classification revealed minor inter-strain differences across cellular components, molecular functions, and biological process categories (Figure 7C). KEGG pathway enrichment identified 886 (PS-JQ08) and 873 (PS-JQ09) genes distributed across 28 metabolic pathways, with 15 pathways containing more than 20 genes. Membrane transport and carbohydrate metabolism predominated in both pathways (Figure 7D). COG analysis annotated 1888 (PS-JQ08) and 1851 (PS-JQ09) proteins, with “General function prediction only” and “Function unknown” as the top two categories in both strains (Figure 7E). CAZy profiling revealed 31 and 29 CAZy-related genes in PS-JQ08 and PS-JQ09, respectively, with glycosyltransferases being the most abundant.

Using Multilocus Sequence Typing (MLST), PS-JQ08 was classified as ST74 under the RIRDC scheme and ST10 under the multi-host scheme, whereas PS-JQ09 was identified as ST346 under the RIRDC scheme but yielded no matching sequence type under the multi-host scheme. We performed whole-genome SNP-based phylogenetic analysis using publicly available complete genomes, and the phylogenetic tree is shown in Supplementary Information, Figure S3.

### 3.6. Analysis of Virulence and Resistance Genes

Comparison with the VFDB-A database identified potential 103 virulence-associated genes in PS-JQ08 and 111 in PS-JQ09 (Supporting Information: Table S5). Both isolates were annotated using virulence factors spanning multiple categories including iron acquisition, lipopolysaccharide (LPS) synthesis, capsule synthesis, adhesion, secretion systems, and antioxidant stress responses. PS-JQ08 harbors various iron acquisition systems (Chu, Hit, HmbR, Isd, and Fbp) and 12 copies of hitC. This indicates the robust iron-acquisition capability of this strain, which is conducive to its survival in the iron-limited environment of the host. The LPS synthesis pathway was remarkably complete, encompassing nearly all biosynthetic steps from lipid A to the core oligosaccharide to the O antigen. The presence of the lsg gene cluster (lsgA/B/D/E/F) suggests a potential role in LPS sialylation, which may contribute to immune evasion. Capsule synthesis genes from diverse sources were also identified, suggesting a potentially complex capsular structure that may contribute to antiphagocytic activity and immune evasion. Multiple adhesion factors were identified, including pili (type IV and Tap type), outer membrane proteins, and adhesion molecules, which may facilitate host colonization and invasion. In addition, genes associated with type III, IV, and VI secretion systems (T3SS, T4SS, and T6SS) were detected, indicating the potential presence of multiple secretion systems capable of delivering effector proteins and modulating host-cell functions.

Based on comparison with the CARD database, a total of 45 potential antimicrobial resistance (AMR)-related genes were identified in strain PS-JQ08 (Supporting Information: Table S6). These genes were associated with resistance to multiple antibiotic classes, including polymyxins (arnA, eptA, ugd, and basS), macrolides (primarily ABC transporter family genes, including macB, msbA, oleC, TaeA, and RanA), sulfonamides (sul4), aminoglycosides (macB and msbA), glycopeptides (vanL, vanR, vanH, and vanT), and tetracyclines [tet(35) and tetA(58)].

Regarding putative resistance mechanisms in PS-JQ08, efflux pump systems (ABC, RND, MFS, MATE families) were the most frequent, followed by target-alteration mechanisms including glycopeptide target modifications (van clusters) and lipopolysaccharide modifications (arnA, eptA, ugd), and enzymatic inactivation mediated by vatB acetyltransferase and NmcR-related β-lactamase regulation. Efflux-related genes accounted for ~52% (24/45) of total putative AMR genes, and target-alteration genes accounted for ~28% (13/45). Detected genes also suggested putative resistance to streptogramins, oxazolidinones, trimethoprim and sulfonamides via target-protection (vgaB, optrA, salE) and target-replacement (dfrA3, sul4) elements.

A total of 43 AMR-related genes were identified in PS-JQ09. Although the two isolates were associated with the same major antibiotic class differences were observed in the specific resistance genes and their abundances. Overall, PS-JQ09 harbors a diverse repertoire of antimicrobial resistance genes, with efflux pump-associated genes and glycopeptide resistance genes being particularly prevalent (Supporting Information: Table S6).

**Figure 8:**
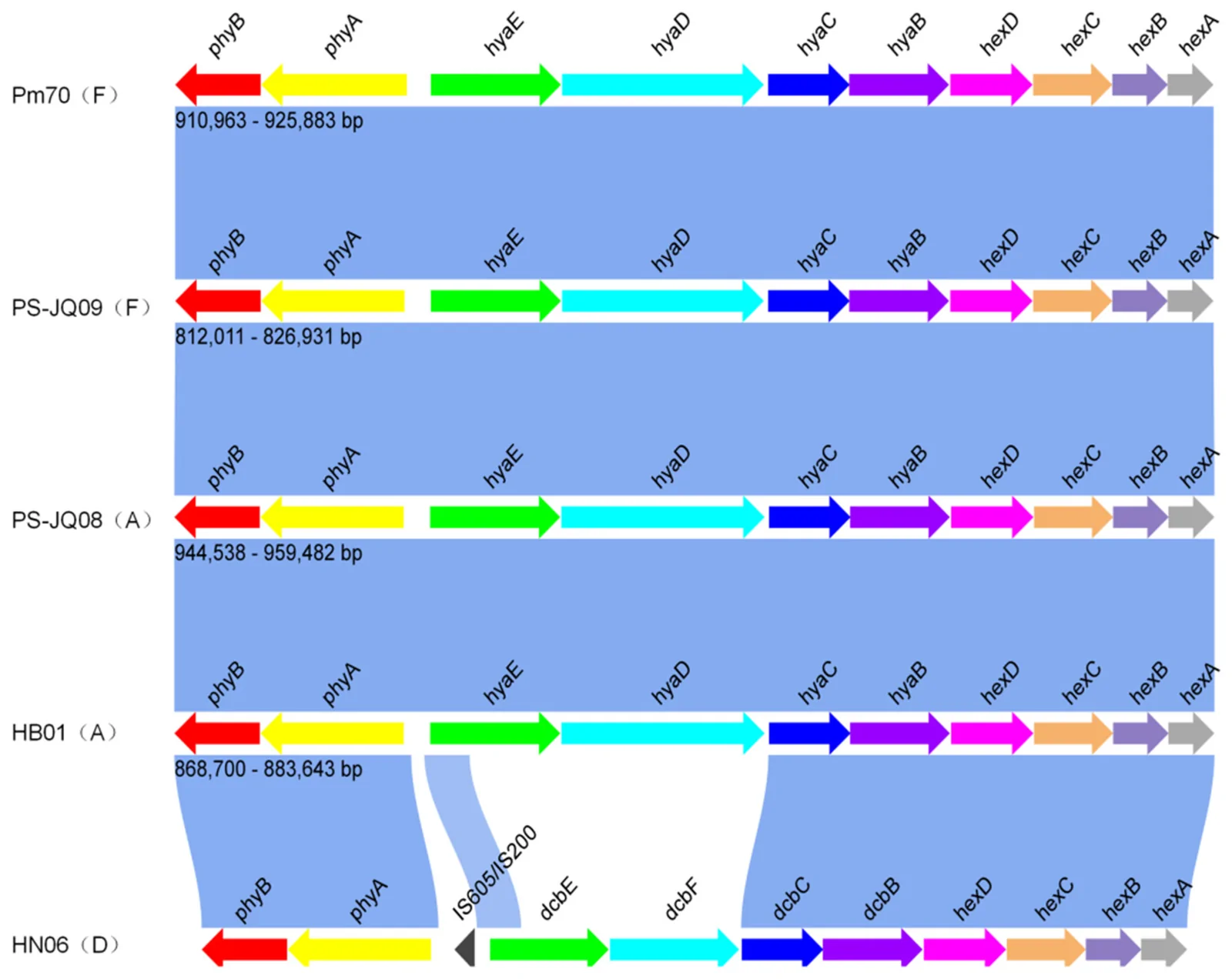
Synteny comparison of the capsule synthesis locus.

Comparative analysis revealed that the capsular locus of PS-JQ08 exhibited high synteny with that of the serogroup A reference strain (HB01), with consistent gene order and intergenic region lengths. In contrast, the capsule locus of PS-JQ09 showed high homology to that of the serogroup F reference strain (Pm70). Notably, the overall architectures of the serogroup A and F capsule loci were relatively similar. However, significant differences were observed in gene composition and transcriptional orientation between the serogroup D and B strains. The results of the synteny comparison are shown in Figure 8.

## 4. Discussion

Pasteurellosis in rabbits is primarily caused by serogroups A, D, and F of *P. multocida*, with serogroup A being the predominant epidemic strain. However, reports of serogroup F infections have shown an increasing annual trend in recent years, and the isolation rate of serogroup F strains from poultry and swine sources in China has increased [18,19]. This epidemiological shift warrants further investigation. Currently available commercial vaccines against *P. multocida* mainly target serogroup A, and existing evidence indicates weak cross-protection between serogroups A and F [20]. Consequently, effective measures for controlling the serogroup F are lacking. Furthermore, the pathogenic mechanisms and genetic characteristics of serogroup F are poorly understood and systematic comparative studies on the pathogenic differences between strains of different serogroups are scarce.

To address these knowledge gaps, this study presents a systematic comparison of the genomic characteristics and pathogenic features of Chinese rabbit-derived *P. multocida* serogroups A and F. Two *P. multocida* isolates representing different serogroups (A and F) were obtained from the same diseased rabbit farm. Using mouse and rabbit models, we confirmed that both isolates exhibited high virulence. Severe clinical signs were observed in mice as early as 6 h after the intraperitoneal challenge, and necropsy revealed severe hemorrhage in the liver and lungs. Similar findings were observed in the rabbit challenge experiments, which demonstrated typical pathological features of *P. multocida* infection. However, distinct pathological differences were observed between the two strains; rabbits challenged with PS-JQ08 (serogroup A) primarily exhibited pulmonary nodules and hepatomegaly, whereas those challenged with PS-JQ09 (serogroup F) showed severe congestion of the liver and lungs. Histopathological examination revealed that the former was mainly characterized by hepatocellular vacuolar degeneration predominated in rabbits infected with PS-JQ08, whereas hepatocellular edema was the predominant lesion in those infected with PS-JQ09. These findings indicate that both isolates are highly virulent but exhibit distinct pathogenic profiles, suggesting that *P. multocida* strains of different serogroups may cause host tissue damage through different pathological mechanisms.

Whole-genome sequencing of the two *P. multocida* isolates completed in this study effectively enriched the reference genome dataset for this species. Comparative genomic analyses of the strains from different hosts and serogroups. Phylogenetic analysis revealed that the swine-origin serogroup A reference strain HB03 and strain 3480 formed a highly supported monophyletic clade with PS-JQ08, indicating a close phylogenetic relationship. In contrast, PS-JQ09 clustered within a distinct evolutionary branch alongside the reported swine-origin serogroup F strain HN07 from abroad, suggesting unique population evolutionary characteristics. Synteny analysis demonstrated greater than 98% nucleotide homology between the two isolates and the reference genomes, indicating a high degree of genomic conservation.

Virulence-factor annotation revealed that the overall virulence profiles of the two strains were similar, encompassing complete functional modules associated with adhesion, invasion, immunomodulation, antiphagocytosis, secretion systems, antioxidant defense, and toxin production. Both strains carried a complete Iron Acquisition Island, a genetic element that may significantly enhance competitive survival under iron-restricted stress conditions within the host [21]. Both strains also possessed a comprehensive antioxidant defense system, including *katA*, *sodB*, *sodCI*, and *msrA/B*. Key outer membrane proteins, including OmpA, OmpP5, OapA, and LptD, were also identified, consistent with previous reports by Li et al. [22,23]. Notably, the core virulence factor of Bordetella pertussis, the bifunctional toxin hemolysin/adenylate cyclase (cyclolysin/ACT), was annotated in the genome of strain PS-JQ08. As a key pathogenic factor, this toxin inhibits the phagocytic and bactericidal functions of host macrophages and neutrophils, thereby promoting bacterial colonization and proliferation in alveolar tissues. It also directly induces pulmonary inflammatory damage by triggering host cell apoptosis [24,25]. The presence of hemolysin/adenylate cyclase toxin, together with three predicted copies of the antioxidant-related *mip* homologue (identified from VFDB-B, Supporting Information: Table S7), may collectively explain the pathological features dominated by hepatocellular vacuolar degeneration observed in this strain. Conversely, the serogroup F strain (PS-JQ09), possessing unique genetic determinants, such as sialyltransferase (cstIII) and the Shu iron uptake system, may mediate a damage pattern characterized by hepatocellular edema through different immune evasion and iron acquisition pathways [21,26]. This pathogenic link remains a hypothesis and requires further functional genetic experiments for validation. This study was performed based on two rabbit-derived *P. multocida* isolates. Caution should be taken when generalizing these results to broader field strains. In addition, the resistance genotypes identified in this study were not experimentally validated against phenotypic antimicrobial susceptibility testing, and such genotype–phenotype correlation should be addressed in future work. Animal challenge experiments were carried out with fixed inoculation doses without LD_50_ measurement or in vivo bacterial load quantification. Further studies with more field isolates and quantitative virulence assays are needed to elaborate the pathogenic mechanisms of serogroup A and F *P. multocida* in rabbits.

## 5. Conclusions

In summary, this study identified two highly virulent rabbit-derived *P. multocida* isolates representing serogroups A and F that caused respiratory disease and mortality in experimental animal models. The complete genomes of the two bacterial strains were assembled. Studies using mouse and rabbit models revealed distinct pathogenic profiles. The key virulence factor, cyclolysin, was identified in serogroup A strain PS-JQ08, which is closely associated with hepatocyte apoptosis. Combined with pathogenicity data obtained from two animal models, these findings advance our understanding of the differential pathogenic characteristics of the strains and lay an important genomic foundation for vaccine development, diagnostic marker screening, and the control of drug resistance in rabbit pasteurellosis.

## Figures and Tables

**Table 1:** Summary of prophage regions in the sequenced *P. multocida* strains.

Strain	Prophage Region	Location	Length	GC%
PS-JQ08	1	35,345–133,431	98,087	39.1
	2	590,229–598,360	8132	34.2
	3	1,035,331–1,073,621	38,291	40.5
	4	1,352,656–1,392,518	39,863	39.8
	5	1,399,888–1,482,299	82,412	40.2
	6	1,651,656–1,695,773	44,118	40.9
	7	2,330,179–2,455,863	125,685	38.4
PS-JQ09	1	34,215–94,828	60,614	37.3
	2	420,815–436,980	16,166	35.1
	3	1,052,590–1,168,697	116,108	40.2
	4	1,274,625–1,333,192	58,568	38.4
	5	1,922,202–1,944,019	21,818	42.2
	6	2,328,437–2,365,944	37,508	38.1

## Data Availability

The data presented in this study are available on request from the corresponding author. The genomic sequencing data of Pasteurella multocida strain PS-JQ08 generated in this study have been deposited in the National Microbiology Data Center (NMDC) under accession number NMDC10020615 (https://nmdc.cn/resource/genomics/project/detail/NMDC10020615, accessed on 10 February 2026) and NMDC10021013 (https://nmdc.cn/resource/genomics/project/detail/NMDC10021013, accessed on 28 September 2026).

## Supplementary Materials

The following supporting information can be downloaded at: https://www.mdpi.com/article/10.3390/vetsci13101058/s1, Table S1: Mortality and sampling schedule of mice in the challenge experiment. Table S2: Mortality and sampling schedule of rabbit in the challenge experiment. Figure S1: Read length distribution of genomic sequencing reads from the two isolates. (A) PS-JQ8, (B) PS-JQ09. Table S3: Sequencing and assembly statistics of the two strains. Table S4: Statistics of gene annotation of strains PS-JQ08 and PS-JQ09 across multiple databases. Figure S2: Gene structure of the genomic islands in the two strains. Figure S3: Whole-genome SNP-based phylogenetic tree constructed using publicly available complete genomes. Table S5: Representative virulence-associated genes predicted by the VFDB-A database, Table S6: Antimicrobial resistance genes of Pasteurella multocida PS-JQ08 and PS-JQ09. Table S7: Representative virulence-associated genes predicted by the VFDB-B database.
