# Supplementary material for: Pathogenicity and Comparative Genomics Analysis: Pasteurella multocida Serogroups A and F Isolated from Domestic Rabbits in China
Source: Vet Sci. 2026 Oct 8;13(10):1058. doi: 10.3390/vetsci13101058 (PMC13709917; doi:10.3390/vetsci13101058)
Supplement: Supplementary file 1 [file vetsci-13-01058-s001.zip › vetsci-4587297-supplementary.pdf]

Table S1. Mortality and sampling schedule of mice in the challenge experiment.(n =5 per group), NC (uninfected control), PS-JQ08 (PS-JQ08-infected), PS-JQ09 (PS-JQ09-infected).

Time (h)	PS-JQ08		PS-JQ09		NC	
	No. of deaths	Histopathology	No. of deaths	Histopathology	No. of deaths	Histopathology
12	3		2			
24	1	yes	1	yes		
36						
48	1	yes	1	yes	1	yes
60						
72			1	yes	4	yes

Table S2. Mortality and sampling schedule of rabbits in the challenge experiment.(n =5 per group), NC (uninfected control), PS-JQ08 (PS-JQ08-infected), PS-JQ09 (PS-JQ09-infected).

Time (d)	PS-JQ08		PS-JQ09		NC	
	No. of deaths	Histopathology	No. of deaths	Histopathology	No. of deaths	Histopathology
2	1					
3	1	yes	1	yes		
4			1			
7	1	yes	1	yes	1	yes
10	1					
11	1	yes				
14			2	yes	4	yes

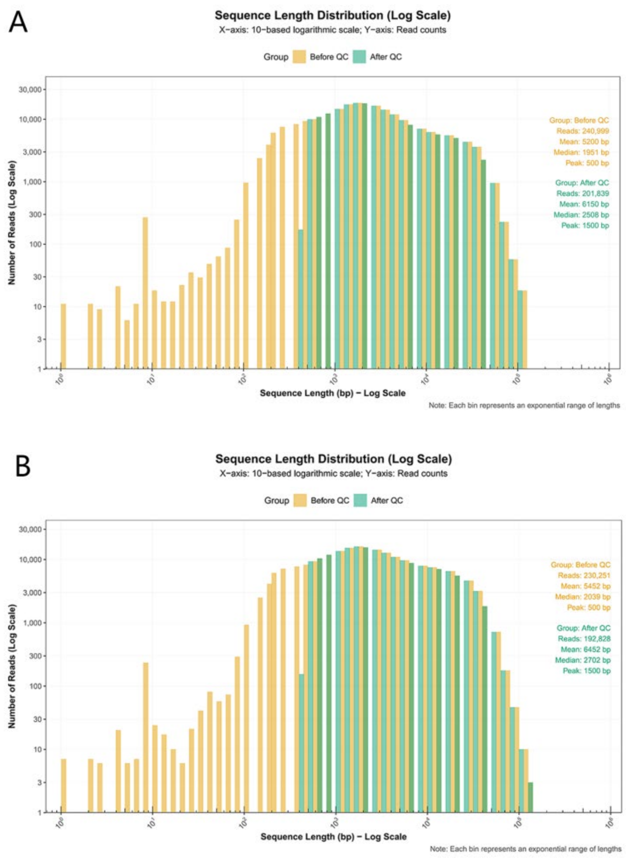

Figure S1. Read length distribution of genomic sequencing reads from the two isolates. (A) PS-JQ08, (B) PS-JQ09.

Table S3. Sequencing and assembly statistics of the two strains.

	**PS-JQ08**	**PS-JQ09**
Clean short-read count	9 111 574	11 021 292
Total clean short-read bases (bp)	1 355 789 277	1 644 260 629
Total assembly size (bp)	2 627 667	2 535 569
Number of contigs	1	1
N50 (bp)	2 627 667	2 535 569
GC content (%)	42.36	42.24
Short-read coverage (×)	352.70	471.03
Completeness assessment-BUSCO Complete (%)	98.39	99.19

Completeness assessment-CheckM completeness (%)	99.54	99.51
Assembly status	Single, complete circular chromosome	Single, complete circular chromosome
Total predicted genes	2 615	2 509
Protein-coding sequences (CDS)	2 492	2 383
rRNA genes	19	20
tRNA genes	56	57
ncRNA genes	4	4
Plasmid detected	No	No

Table S4. Statistics of gene annotation of strains PS-JQ08 and PS-JQ09 across multiple databases.

**Database**	**PS-JQ08**		**PS-JQ09**	
	**Number of genes**	**Percentage(%)**	**Number of genes**	**Percentage(%)**
Annotated in NR	2491	99.96	2380	99.87
Annotated in COG	1888	75.76	1851	77.68
Annotated in CDD	1066	42.78	1053	44.19
Annotated in PFAM	1747	70.1	1697	71.21
Annotated in GO	486	19.5	485	20.35
Annotated in KEGG	886	35.55	873	36.63
Annotated in CAZy	31	1.24	29	1.22
Annotated in TCDB	367	14.73	345	14.48
Annotated in PHI	561	22.51	547	22.95
Annotated in VFDB_A	147	5.9	143	6
Annotated in VFDB_B	254	10.19	248	10.41
Annotated in CARD	59	2.37	59	2.48
Annotated in BactMet_exp	104	4.17	104	4.36
Annotated in BactMet_pre	125	5.02	126	5.29
Annotated in SARG	3	0.12	3	0.13
Annotated in at least one database	2491	99.96	2380	99.87
Annotated in all database	0	0	0	0
Total genes	2492	100	2383	100

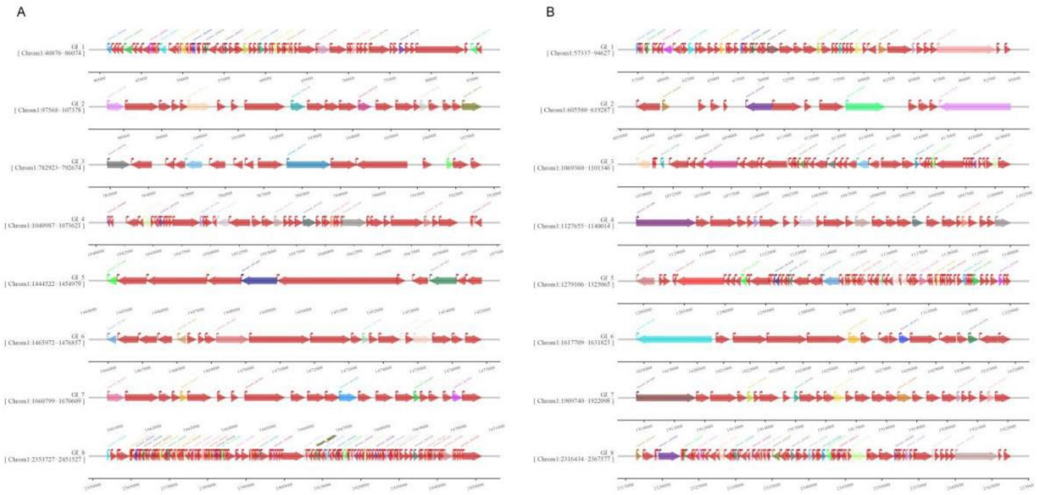

Figure S2. Gene structure of the genomic islands in the two strains. A: The genomic islands of PS-JQ08; B: The genomic islands of PS-JQ09.

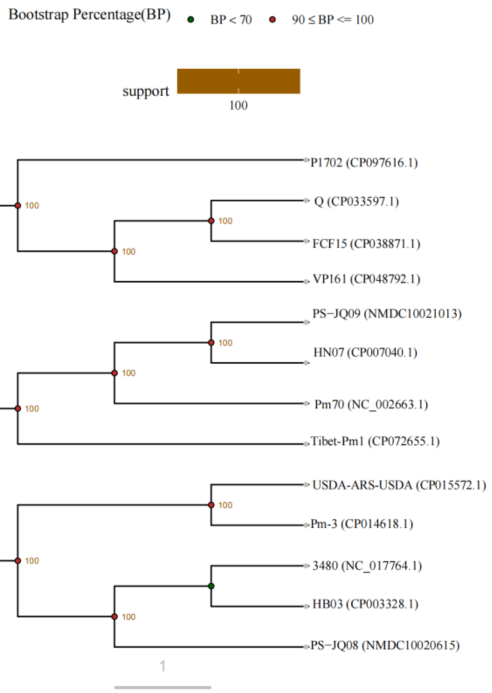

Figure S3. Whole-genome SNP-based phylogenetic tree constructed using publicly available complete genomes.

Table S5. Representative virulence-associated genes predicted by the VFDB-A database.

**Function**	**Virulence Factors**	**PS-JQ08 Related Genes**	**PS-JQ09 Related Genes**
Adherence	IlpA	IlpA	IlpA
	MAM7	VP1611	VP1611

	Alginate	algU	algU
	Flp type IV pili	flpF	flpF
	Lap	lap	lap
	LpeA	lpeA	lpeA
	OapA	oapA	oapA
	OmpA	ompA	ompA
	P5 protein	ompP5	ompP5
	Type IV pili	pilE, pilF, pilR	pilE, pilF, pilR
	Tap type IV pili	tapB, tapQ	tapB, tapQ
	Hsp60	htpB	htpB
	Autoinducer-2	luxS	luxS
	ClpP	clpP	clpP
	Flagella (regulatory)	fleR/flrC	fleR/flrC
Invasion	LPS	fabZ, acpXL, kdtB	fabZ, acpXL, kdtB
	LOS	rfaD, rfaE	rfaD, rfaE
	Chu	chuV	chuV
	Capsule	lipA	lipA
Immune modulation	LOS	acpXL, bplF, bplL, fabZ, galE, galU, gmhA/lpcA, htrB, kdkA, kdsA, kdsB, kdtA, kdtB, lgtA (2), lgtC, lgtF, lic2A, lpxA, lpxB, lpxC, lpxD, lpxH, lpxK, lsgA, lsgB, lsgD, lsgE, lsgF, msbA, msbB, neuA, opsX/rfaC, orfM, rfaD (2), rfaE, rfaF, rffG, waaC, waaQ, wbaP/rfbP, wecA, yhxB/manB, kpsF	acpXL, bplF, fabZ, galE, galU, gmhA/lpcA, htrB, kdkA, kdsA, kdsB, kdtA, kdtB, lgtC, lgtF, lic2A, lpxA, lpxB, lpxC, lpxD, lpxH, lpxK, lsgA, lsgB, lsgD, lsgE, lsgF, msbA (2), msbB, neuA, opsX/rfaC, orfM, rfaD, rfaE, rfaF, rffG, waaC, waaQ, wbaP/rfbP, wecA, yhxB/manB, kpsF, cstIII
Motility	—	—	—
Antiphagocytosis	Capsule	bexA, bexC, bexD, cap8E, cap8F, cap8G, cps4F, cpsA, cpsB, ctrC, hscB, kpsF, lipA, tviB, wcbN	bexA, bexC, bexD, cap8E, cap8F, cap8G, cps4F, cpsA, cpsB, ctrC, hscB, kpsF, lipA, tviB, wcbN
Iron uptake	Chu system	chuU (4), chuV	chuU (3), chuV
	Hgp system	hgpC	hgpC
	Hhu system	hhuA (2)	hhuA

	Hit system	hitC (12)	hitC (15)
	HmbR	hmbR	hmbR
	Isd system	isdF	isdF
	Fbp system	fbpC (2)	fbpC
	Fep system	fepC	fepC
	Fur regulation	fur	fur
	Shu system	—	shuU
	Pyoverdine synthesis	pvcC	pvcC
Biofilm formation	Flagella	fleR/flrC	fleR/flrC
	Alginate	algU	algU
Secretion systems	T3SS	cdsN, ssaN	cdsN, ssaN
	T4SS (Dot/Icm)	lirB, lpg2936	lirB, lpg2936
	T6SS (HSI-I)	ppkA, tagR, tagS, tagT	ppkA, tagR, tagS, tagT
Antioxidant	Catalase	katA	katA
	Superoxide dismutase	sodB, sodCI	sodB, sodCI
	Methionine sulfoxide reductase	msrA/B(pilB)	msrA/B(pilB)
	Neutrophil activating protein	napA	napA
Toxin	Hemolysin	hlyA, hlyB (2), cylA (2), cylG	hlyA, hlyB (2), cylA (2), cylG
	RTX toxin	rtxE	rtxE
	Cyclolysin	cyaB	—
Other	Magnesium transport	mgtC	mgtC
	Hexose phosphate transport	hpt (2)	hpt
	Stringent response	relA	relA
	DNA repair	recN	recN
	Fatty acid efflux	farB	farB
	Lipoprotein signal peptidase	lspA	lspA
	Phosphoethanolamine transferase	eptC	eptC
	Two-component regulator	bprB, pilR	bprB, pilR
	Mucoidy regulation	algW, mucD, mucP	algW, mucD, mucP
	Cytochrome c maturation	ccmA, ccmB, ccmC, ccmE, ccmF (2)	ccmA, ccmB, ccmC, ccmE, ccmF (2)

	Sugar-binding regulator	bopD	bopD

Table S6. Antimicrobial resistance genes of *Pasteurella multocida* PS-JQ08 and PS-JQ09.

**Resistance Category**	**ARO_Name**	**PS-JQ08**	**PS-JQ09**
Aminoglycoside resistance	Acinetobacter baumannii AbaF	+	+
	efrA	+	+
	TaeA	+	+
Beta-lactam resistance	Chlamydia trachomatis intrinsic murA conferring resistance to fosfomycin	+	+
	LptD	+	+
	NmcR	+	+
	OmpA	+	+
Fluoroquinolone resistance	cpxA	+	+
	CRP	+	+
	Pseudomonas aeruginosa CpxR	+	+
	RanA	+	+
	salE	+	+
	smeR	+	+
	TxR	+	+
Glycopeptide resistance	vanH gene in vanA cluster	+	+
	vanH gene in vanM cluster	+	+
	vanL	+	+
	vanR gene in vanE cluster	+	+
	vanR gene in vanO cluster	+	+
	vanT gene in vanG cluster	+	+
Macrolide/lincosamide/streptogramin resistance	macB	+	+
	MexH	+	+
	MexW	+	+
	oleC	+	+
	optrA	+	+
	vatB	+	+
	vgaB	+	+
Multidrug resistance efflux pumps	baeR	+	+

	basS	+	+
	clbB	+	+
	farB	+	+
	H-NS	+	-
	hmrM	+	-
	msbA	+	+
	YajC	+	+
Peptide antibiotic resistance	arnA	+	+
	eptA	+	+
Phenicol resistance	bcr-1	+	+
Rifampicin resistance	Bifidobacterium adolescentis rpoB mutants conferring resistance to rifampicin	+	+
Sulfonamide resistance	sul4	+	+
Tetracycline resistance	tet(35)	+	+
	tetA(58)	+	+
Lipopolysaccharide modification	LpsA	+	-
	ugd	+	+
Other/Unclassified	rsmA	-	+
	dfrA3	+	+

Table S7. Representative virulence-associated genes predicted by the VFDB-B database.

**Function**	**Virulence Factors**	**PS-JQ08 Related Genes**	**PS-JQ09 Related Genes**
Adherence	IlpA	IlpA	IlpA
Adherence	ClpP	clpP	clpP
	Streptococcal lipoprotein rotamase A	slrA	slrA
	Trigger factor	tig/ropA	tig/ropA
	Streptococcal enolase	eno	eno
	Listeria adhesion protein	lap	lap
	OapA	oapA	oapA
	P5 protein	ompP5	ompP5
	Autoinducer-2	luxS	luxS
	OmpA	ompA	ompA
	Fibronectin-binding protein	comE1,ligA	comE1,ligA
	NlpI	ECS88_3547	ECS88_3547
	MOMP	CT396(2)	CT396(2)

	MAM7	VP1611	VP1611
	IlpA	IlpA	IlpA
	Hsp60	htpB	htpB
Immune modulation	LPS	kdtB,fabZ,acpXL,hisF	kdtB,fabZ,acpXL,hisF
	LOS	rfaE,rfaD(2),CFF8240_1412,CJJ81176_1162,kdsB,msbA(2),opsX/rfaC(2),kdtA,lpxH,lsgB,lpxK,msbB,lgtC,orfM,kdkA,galE,waaQ,lic2A,lgtF,yhxB/manB,lex2B,galU,rffG,lpxD,yhbX(2),lpxB,lpxA,rfaF,lpxC,gmhA/lpcA,neuA,htrB,kdsA,lgtA(2),kpsF,lsgF,lsgE,lsgD,lsgA,wecA	rfaE,rfaD,CFF8240_1412,CJJ81176_1162,kdsB,msbA(2),opsX/rfaC(2),kdtA,lpxH,lsgB,lpxK,msbB,lgtC,orfM,kdkA,galE,waaQ,lic2A,lgtF,yhxB/manB,lex2B,galU,rffG,lpxD,lpxB,lpxA,rfaF,lpxC,gmhA/lpcA,neuA,htrB,kdsA,kpsF,lsgF,lsgE,lsgD,lsgA,wecA
	Exopolysaccharide	mrsA/glmM,pgi,manA	mrsA/glmM,pgi,manA
	Phosphoethanolamine modification	lptA	lptA
Motility	Flagella	fliI	fliI
	Polar flagella	flmH	flmH
Antiphagocytosis	Alginate	algU	algU
	Capsule	hcsA,bexA,bexC,bexD,hscB,cpsB,oppF(3),ctrC,BJAB07104_00090,ABBFA_003450,ABBFA_003451,ABAYE3808,fnlA,ABTJ_03752,ABZJ_00088,uppS	hcsA,bexA,bexC,bexD,hscB,cpsB,oppF(3),ctrC,BJAB07104_00090,ABBFA_003451,ABAYE3808,ABTJ_03752,uppS
	O-antigen	YPA_2067	YPA_2067
	Capsular polysaccharide	wza,wzb,wzc	wza,wzb,wzc
	LPS O-antigen(P. aeruginosa)	hisH2,PSPA7_1983	hisH2,PSPA7_1983
	Capsule I	wcbN	wcbN
Iron uptake	HitABC	hitC(2)	hitC(5)
	Fur	fur	fur
	CcmC	ccmC	ccmC
	Ton system	exbB,exbD	exbB,exbD
	Iron/managanease transport	sitB,sitC,sitA,sitD	sitB,sitC,sitA,sitD
	Haemophilus iron transport locus	hitA,hitB,hitC(5)	hitA,hitB,hitC(4)

	Heme biosynthesis	hemB,hemC,hemD,hemG,hemN(2),hemX,hemA,hemE,hemH,hemL,hemM,hemY,hemR	hemB,hemC,hemD,hemG,hemN(2),hemX,hemA,hemE,hemH,hemL,hemM,hemY,hemR
	Hemoglobin and hemoglobin-haptoglobin binding proteins(Hgps)	hgpB,hgpC	hgpB,hgpC
	Mg2+ transport	mgtC	mgtC
	ABC-type heme transporter	hmuV	hmuV
	ciu iron uptake and siderophore biosynthesis system	ciuD	ciuD
	Hemin uptake	chuU	chuU
	ABC transporter	fbpC	fbpC
	Haemoglobin receptor	hmbR	hmbR
	Transferrin and lactoferrin-mediated iron uptake	ctuA	ctuA
	direct heme uptake system	chuC,hemU(2),hemT,hutZ(2),huvX	chuC,hemU(2),hutZ(2),huvX
	pyoverdine	pvcC	pvcC
	Chu	chuV	chuV
	vibrioferrin	pvuE(2),pvuD,pvuC,pvuB	pvuE(2),pvuD,pvuC
	vulnibactin	VVA1298(2)	VVA1298(2)
	thioquinolobactin	qbsC	qbsC
	HhuA	hhuA	
Biofilm formation	BopD	bopD	bopD
	Alginate regulation	mucP,algW,mucD	mucP,algW,mucD
Secretion systems	Accessory secretion factor	secA2	secA2
	Type IV pili	pilD,comE/pilQ	pilD,comE/pilQ
	P. syringae TTSS effectors	hopL1,hopAJ2,hopAN1	hopL1,hopAJ2,hopAN1
	Hcp secretion island-1 encoded type VI secretion system(H-T6SS)	Psyr_2626,PSPTO_2876,Psyr_2628,ppkA	Psyr_2626,PSPTO_2876,Psyr_2628,ppkA
	T4SS effectors	CBUD_0561	CBUD_0561
	TSS-5	regB	regB
	T3SS	esrB	esrB

	Cpi-1a + Cpi-1(SPI-1 like)	armR	armR
	Cpi-2 encoded T3SS(SPI-2 like)	csrB,csrA	csrB,csrA
	LspA1-LspB	HD1155	HD1155
	HrpA-HrpB	NMB1780	
	type IV pili	vfr,ptfA,hofB,hofC,pilF	vfr,ptfA,hofB,hofC,pilF
	fimbrial low-molecular-weight protein(Flp) pili	flp-1,tadV,rcpC,rcpA,rcpB,tadZ,tadA,tadB,tadD,tadE,tadF,tadG	flp-1,tadV,rcpC,rcpA,rcpB,tadZ,tadA,tadB,tadC,tadD,tadE,tadF,tadG
	Dot/Icm	lpg2936	lpg2936
Antioxidant	HP-NAP	napA	napA
	SodCI	sodCI	sodCI
	SodB	sodB	sodB
	AhpC	ahpC	ahpC
	Catalase	katA	katA
	Methionine sulphoxide reductase	msrA/B(pilB)	msrA/B(pilB)
Toxin	Phytotoxin phaseolotoxin	argD,argK	argD,argK
	Phospholipase D	plcD	plcD
	Hemolysin, HlyA	hlyA	hlyA
	The repeat in toxin(RTX)	rtxE	rtxE
	colibactin	clbG	clbG
Other	Streptococcal plasmin receptor/GAPDH	plr/gapA	plr/gapA
	Lipoprotein-specific signal peptidase II	lspA	lspA
	FAS-II	kasB	kasB
	Purine synthesis	purC	purC
	Leucine synthesis	leuD	leuD
	(p)ppGpp synthesis and hydrolysis	relA	relA
	Cytochrome c muturation(ccm) locus	ccmA,ccmB,ccmC,ccmE,ccmF(2)	ccmA,ccmB,ccmC,ccmE,ccmF(2)
	Alternative sigma factor RpoS	rpoS	rpoS
	N-linked protein glycosylation	pglJ	
	Type IV pili biosynthesis	pilR	pilR
	GacS/GacA two-component system	gacA(2)	gacA

	Proline synthesis	proC	proC
	Tryptophan synthesis	trpD	trpD
	Sigma A	sigA/rpoV	sigA/rpoV
	Glutamine synthesis	glnA1	glnA1
	Mycobactin	mbtI(2)	mbtI(2)
	MymA operon	fadD13	
	Trehalose-recycling ABC transporter	sugC(5)	sugA,sugB,sugC(6)
	Copper exporter	ctpV	ctpV
	Nucleoside diphosphate kinase	ndk	ndk
	Tyrosine phosphatase	ptpA	ptpA
	SenX3	senX3	senX3
	RegX3	regX3	regX3
	Sugar-uptake system	hpt(2)	hpt
	Oligopeptide-binding protein	oppA	oppA
	ABC transporter for dispersin	aatC	aatC
	Opacity protein	opa	opa
	FarAB	farB	farB
	MtrCDE	mtrD	mtrD
	Recombinational repair protein	recN	recN
	Mip	mip(3)	mip
